# Systematic review of screening tools for common soccer injuries and their risk factors

**DOI:** 10.4102/sajp.v77i1.1496

**Published:** 2021-02-12

**Authors:** Raphael Christopher, Corlia Brandt, Natalie Benjamin-Damon

**Affiliations:** 1 Department of Physiotherapy, Faculty of Health Sciences, University of the Witwatersrand, Johannesburg, South Africa

**Keywords:** common soccer injuries, common injury risk factors, screening tools prevention and prediction, screening tools accuracy, sensitivity and specificity

## Abstract

**Background:**

Several screening tools are available for use in a clinical setting to predict injury. However, there is a lack of evidence regarding the accuracy of these tools to predict soccer-specific injuries.

**Objectives:**

The purpose of this systematic literature review was to determine the psychometric properties or accuracy of screening tools for common soccer injuries.

**Methods:**

A systematic review of diagnostic test accuracy was undertaken based on the Joanna Briggs Institute (JBI) procedure for conducting systematic reviews. Databases such as SPORT Discus, Cinahl, Medline, Science Direct, PubMed and grey literature were searched in order to access suitable studies.

**Results:**

A total of 10 studies were included for the analysis – three were analysed quantitatively whilst the remaining seven were analysed qualitatively. The screening tools were of high reliability, sensitivity and specificity (calculated as intraclass correlation coefficient [ICC] (0.68 95% confidence interval [CI]: 0.52–0.84 and 0.64 95% CI: 0.61–0.66, respectively).

**Conclusion:**

The screening tools assessed for the prediction of common soccer injuries that emerged from this systematic review include the Functional Movement Screening (FMS™), the Landing Error Scoring System (LESS), the Tuck Jump Assessment, the Soccer Injury Movement Screening (SIMS) and the conventional hamstrings to quadriceps ratio; all with good evidence of predicting common soccer injuries. These tools were of high sensitivity and specificity thus reliable for soccer screening.

**Clinical implications:**

The validity of these tools is acceptable and therefore the authors recommend that these tools be included in an injury prevention programme for soccer players.

## Introduction

Van Mechelen et al.’s sequence of prevention model states that by constant assessment, injury mechanisms and risk factors are identified, highlighting the fact that collecting and recording of data is a core approach for preventing injuries (Van Mechelen, Hlobil & Kemper 1992). A similar proposal states that assessing epidemiological information in teams with similar characteristics, initiating management plans, estimating and assessing risk may help prevent injuries (Clarsen, Myklebust & Bahr 2012; Fuller et al. 2007). Several screening tools are available for use in the clinical setting; these include: the Functional Movement Screening (FMS™) tools, Landing Error Scoring System (LESS), Y-Balance Test, Star Excursion Balance Test, Drop Jump Screening Test and the Tuck Jump Analysis (Chimera & Warren 2016; Lai et al. 2017). These screening tools only recently received researchers’ attention, hence there is a dearth of data regarding their applicability, validity and reliability (Chimera & Warren 2016). For example, the Y-Balance Test (YBT) used alone is reported by Lai et al. (2017) as not being capable of predicting injury to the lower extremities and recommends caution by rehabilitation professionals in its use as a standalone screening tool for injuries. However, the YBT is touted as being capable of identifying at-risk soccer players when included in physical examinations (Gonell, Aurelio & Romero 2015).

Several systematic reviews related to common soccer injuries have been conducted mostly in high-income countries. However, none of them addressed the screening tools for common soccer injuries. The focus of the reviews was on the risk factors for hamstring and groin injuries (Foreman et al. 2006; Maffey & Emery 2007; Shadle & Cacolice 2016; Van Beijsterveldt et al. 2013). The only review that explores the screening tools to predict injury to the lower extremities is not specific to soccer as it involved a wide range of team sports, namely hockey, football, soccer, volleyball and basketball (Dallinga, Benjaminse & Lemmink 2012).

The systematic review of Dallinga et al. (2012) on the screening apparatus for lower limb injuries, did however make a clinical contribution to recognise tests, which may foresee injuries in sport by employing a search technique that comprised articles from the nineties. They followed a set of incorporated criteria and analysed methodological positioning.

A need to conduct a systematic review to establish the validity and reliability of screening tools in soccer was therefore identified. This was further supported by a statement by Bahr (2016) who reports scepticism about the effectiveness of risk factor screening tools in predicting injury to a satisfactory level of accuracy for reducing injury risk, mainly because of a lack of research evidence.

The validity and reliability (accuracy) of screening tools used in soccer, in the context of soccer-specific epidemiology and risk factors, therefore need to be established to determine the effectiveness of their clinical use. Applicability can then be determined by the evaluation of the tests’ properties in pertinent populations using factual statistical tools (Bahr 2016). The purpose of our study was to conduct a systematic review on screening tools for common soccer injuries and the accuracy of the available screening tools to determine injuries in soccer players.

## Method

Our systematic review of diagnostic test accuracy was performed based on the Joanna Briggs Institute (JBI) guidelines (Campbell et al. 2020) (see Appendix 1) with a meta-analysis (forest plot) to increase analytic precision in the quantitative systematic reviews. The population involved was soccer players, male and/or female. The context entailed the inclusion of professional, elite and social players. The context of the studies included screening tools and accuracy of screening tools. The index tests included screening tools for common soccer injuries, whilst the outcomes referred to the validity and reliability of the included tests. Non-English studies, those published before 2000, and animal studies, were excluded. Observational, prospective and retrospective cohort, case-control, cross-sectional studies, case series and case reports, as well as clinical studies such as randomised clinical trials and other comparative studies were included.

### Data sources and searches

In order to access the relevant literature for our review, the search procedure was accomplished by three distinct steps:

- Step 1: involved the selection of keywords from the abstract and title of our study.
- Step 2: involved a comprehensive search strategy and the use of specific keywords to provide relevant index terms amenable to the included databases.
- Step 3: involved selection of relevant studies found, based on their abstracts, titles and limitation by date. Databases such as SPORT Discus, Cinahl, Medline, Science Direct, PubMed and grey literature were accessed. Key terms included:
  - (Screening OR ‘functional screening’ OR ‘functional movement screening’ OR ‘FMS™’ OR ‘injury screening’) AND (‘elite soccer players’ OR ‘elite football players’) AND (‘soccer injuries’ OR ‘football injuries’). These keywords were used for screening tools for common soccer injuries.
  - (Accuracy OR validity OR reliability OR sensitivity OR specificity) AND (soccer OR football) were used for the accuracy of screening tools for common soccer injuries.

### Selection of studies and methodological quality

Relevant studies were selected based on their abstracts and titles. It also involved critical appraisal of the selected studies using the JBI quality appraisal tool (Campbell et al. 2020). The items of the appraisal tool used consists of study, author, sample type, method of recruiting participants, sample size, settings, analysis, outcome measurement, measurement validity, statistical analysis, important differences accounted for, subpopulation objective criteria and the decision for inclusion or exclusion with an additional option of providing further information. The selection of studies and the critical appraisal of the studies were conducted blindly by the first author and an assistant (RC and JA). Inconsistencies in the data reviewed were resolved through discussion with the second author (CB).

### Risk of bias

The risk of bias (ROB) was assessed by the three authors and followed the guidelines stated in the risk of bias of Non-Randomised Studies (ACROBAT-NRSI version 1.0.0) tool. The ROB assessment for each study has seven domains.

The domains are described as: bias because of confounding, selection bias of participation, bias in measurement of interventions, bias because of departures from intended interventions, missing data bias, outcome measurement bias and selective reporting bias. These domains were judged using the ‘low’, ‘moderate’, ‘serious’ and ‘critical’ scale.

### Grade of evidence

The grade of evidence was based on 10 recommended key items (Mueller et al. 2018). Ten key recommendations were observed for the class of evidence for the review, namely protocol development, research question, search strategy, study eligibility, data extraction, study designs, risk of bias assessment, publication bias, heterogeneity and statistical analysis. Five domains (risk of bias, inconsistency, indirectness, impression and publication bias) as described by Guyatt et al. (2008) were also considered to determine the confidence in the overall estimates and recommendations.

### Data extraction

We used a standardised data extraction tool to extract the significant data from the included articles. This was independently undertaken by the first author (RC) and an assistant (JA) and unresolved inconsistencies were resolved by the second author (CB). The data extracted included social, demographic, seasonal and other factors, dates of survey or intervention, definitions of conditions and populations, inclusion and exclusion criteria, mean age, sex, sample size, statistical methods used to analyse data in the identified studies, specificity and sensitivity.

### Data analysis

Data were analysed using Stata statistical software, version 15.1. Incidence rates and odds ratios, and sensitivity and specificity were analysed with their respective 95% confidence intervals. *I* ^2^ statistic was used to determine the proportion of variation across studies. The forest plot area summarises the results of the test for heterogeneity that was performed.

## Results

### Characteristics of included studies

Database searches returned a total of 95 related studies. Of these 95 studies, 24 were duplicates, whilst 17 studies were retrieved for inclusion based on the selection criteria, population, context and outcome (Figure 1). Fifty-four studies did not meet the population, context and outcome of our review and seven full text studies were excluded as shown in Table 1. Ten studies were therefore included in our review. The characteristics of these studies are shown in Table 2.

**TABLE 1:** Excluded studies.

Studies	Reasons for exclusion
Hartley 2016	Not specific to the population, context and outcomes.
Amin 2013	Not specific to the population and context.
Lintenstein et al. 2014	Not specific to the context.
Myer et al. 2010	Not specific to soccer alone, involves other sports.
Chalmers et al. 2016	Not specific to soccer alone, involves other sports.
Smith et al. 2017	Not specific to soccer alone, involves other sports.
Armstrong & Greig 2018	Not specific to the population.

**TABLE 2a:** Characteristics of the ten included studies.

Screening tools	Population	Intervention	Outcome	Setting	Location	Design
Silva et al. 2017	22 under 16 national competitive soccer players. 2 days.	Screening tools for common soccer injuries.	Anthropometrics. FMS™: the deep squat, hurdle step, in-line lunge shoulder mobility, active straight leg raise, trunk stability push-up and rotary stability and three clearing examinations. Jump performance, instep kick speed (shot speed) and anaerobic performance. Screening tools for: Physical performance	Melgaço School of Sports and Leisure biomechanics laboratory.	Portugal	Observational study
Lehance et al. 2019	57 elite and junior elite male soccer players from a Belgian First Division team.	Screening tools for common soccer injuries.	Functional performance: squat jump and 10m sprint. Screening tools for: the risk of imbalance and implement antagonist strengthening in lower limb. Acute muscles injuries.	Soccer field	Belgium	Observational study
Hammes et al. 2016	238 veteran footballers of 18 teams. 9 months.	Screening tools for common soccer injuries.	FMS™ score Screening tools for: musculoskeletal injuries	Soccer field	Norway	Prospective study
Frohm et al. 2012	18 male elite soccer players of two elite soccer teams. One month.	Screening tools for common soccer injuries.	Functional movement screen: one-legged squat, two-legged squat, and straight leg raise test, and seated rotation test. In-line lunge test, and active hip flexor test. Screening tools for: stability and mobility of the lower limb, Overuse and acute injuries.	Test room	Sweden	Reliability study

FMS, Functional Movement Screening.

**FIGURE 1:**
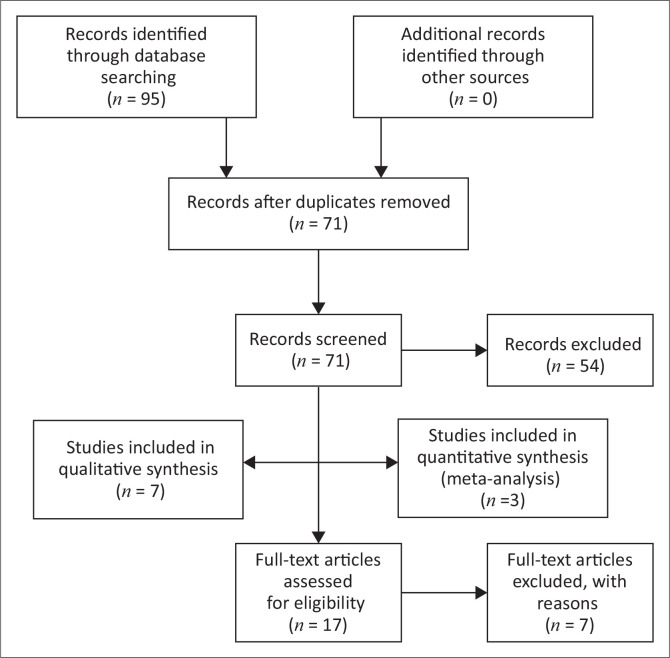
PRISMA flow diagram, Moher et al. (2009).

**TABLE 2b:** Characteristics of the ten included studies.

Screening tools accuracy	Population	Intervention	Outcome	Setting	Location	Design
Padua et al. 2015	829 elite-youth soccer athletes, boys and girls from North Carolina and from Maryland 2006–2009.	Screening tools for common soccer injuries and its accuracy	Less score Screening tools for: Anterior Cruciate Ligaments	Field-based functional movement screening performed at soccer	USA	Cohort study
Chorba et al. 2010	38 female student-athletes.	Screening tools for common soccer injuries and its accuracy	FMS™ score Screening tools for: Anterior Cruciate Ligaments	Field	USA (Ohio)	Cohort study
Read et al. 2017	25 youth soccer players from the academy of a professional English Championship soccer club.	Screening tools for common soccer injuries and its accuracy	Tuck jump Screening tools for: Neuromuscular control Ankle and knee sprain Anterior Cruciate Ligaments	Soccer field	England.	Re-test study
Gabbe et al. 2004	15 participants (9 female and 6 male) volunteered For this study, all participants were staff or postgraduate students of the School of Physiotherapy at the University of Melbourne who reported the absence of a current musculoskeletal injury of the lumbar spine or lower limb	Screening tools for common soccer injuries and its accuracy	Musculoskeletal screening tests: Sit and reach test – risk for hamstring Lumbar spine extension ROM – risk of knee injury. Active hip internal ROM. Active hip external ROM. Passive straight leg raise. Active knee extension test. Active slump test.	Field	Australia	Cohort study
Grykorowicz et al. 2017	66 professional soccer players of the Polish Premier League. 2010–2016	Screening tools for common soccer injuries and its accuracy	Cut-off values for conventional hamstrings-to-quadriceps ratio. Screening tools for: risk of hamstring, Muscle strain or ligament rupture.	Field	Poland	Retrospective study
McCunn et al. 2017	25 healthy, recreationally active university students.	Screening tools for common soccer injuries and its accuracy	Soccer injury movement screen: the anterior reach, single-leg deadlift, in-line lunge, single-leg hop for distance and tuck jump.	Field	Germany	Test-retest design

FMS, Functional Movement Screening; US, United States.

### Risk of bias

The overall judgements showed that out of 10 studies, 7 studies (70%) were graded low risk and 3 studies (30%) were graded moderate risk. One study (Silva et al. 2017) showed bias in confounding, whilst Hammes et al. (2016) also showed selection bias. The source of funding was not reported by any study (Table 3).

**TABLE 3a:** Risk of bias summary table.

Included studies	Bias because of confounding	Risk of bias domains						Overall ROB judgment
		Selection bias of participation	Bias in measurement of interventions	Bias because of departures from intended interventions	Missing data bias	Outcome measurement bias	Selective reporting bias	
Silva et al. 2017	Moderate	Low	Low	Low	Low	Low	Low	Moderate
Lehance et al. 2009	Low	Low	Low	Low	Low	Low	Moderate	Moderate
Hammes et al. 2016	Low	Moderate	Low	Low	Moderate	Low	Low	Moderate
Frohm et al. 2012	Low	Low	Low	Low	Low	Low	Low	Low

ROB, risk of bias.

**TABLE 3b:** Risk of bias summary table.

Included studies	Bias because of confounding	Risk of bias domains						Overall ROB judgement
		Selection bias of participation	Bias in measurement of interventions	Bias because of departures from intended interventions	Missing data bias	Outcome measurement bias	Selective reporting bias	
Padua et al. 2015	Low	Low	Low	Low	Low	Low	Low	Low
Chorba et al. 2010	Low	Low	Low	Low	Low	Low	Low	Low
Read et al. 2017	Low	Low	Low	Low	Low	Low	Low	Low
Gabbe et al. 2004	Low	Low	Low	Low	Low	Low	Low	Low
Grygorowicz et al. 2017	Low	Low	Low	Low	Low	Low	Low	Low
McCunn et al. 2017	Low	Low	Low	Low	Low	Low	Low	Low

ROB, risk of bias.

### Methodology quality

This checklist has three scoring systems, namely, include, exclude or seek further information. The items of the checklist are sample size, sample type, aim, follow-up duration, dependent variables, outcome, outcome measurements, data analysis and identification of objectives. ‘Yes’ or ‘No’ was used to tell if one of the items on the checklist was present or not. A study with a score less than seven was excluded. Of 17 full texts studies, only 10 met the score for inclusion as seen in Table 4.

**TABLE 4:** Methodological rating of included studies.

Screening tools	Sample type	Sample size	Aim	Follow up duration	Dependant variable	Outcome	Outcome measurement	Data analysis	Identification of objective	Results
Amin 2013	Yes	No	Yes	No	Yes	Yes	No	No	Yes	Exclude
Hartley 2016	Yes	No	Yes	No	Yes	Yes	No	No	Yes	Exclude
Silva et al. 2017	Yes	Yes	No	Yes	Yes	Yes	Yes	Yes	Yes	Include
Lehance et al. 2009	Yes	Yes	Yes	Yes	Yes	Yes	Yes	Yes	Yes	Include
Hammes et al. 2016	Yes	Yes	Yes	Yes	Yes	Yes	Yes	Yes	Yes	Include
Frohm et al. 2012	Yes	Yes	Yes	Yes	Yes	Yes	Yes	Yes	Yes	Include
Padua et al. 2015	Yes	Yes	Yes	Yes	Yes	Yes	Yes	Yes	Yes	Include
Chorba et al. 2010	Yes	Yes	Yes	Yes	Yes	Yes	Yes	Yes	Yes	Include
Myer et al. 2010	No	No	No	No	No	No	No	No	No	Excluded
Read 2017	Yes	Yes	Yes	Yes	Yes	Yes	Yes	Yes	Yes	Include
Gabbe et al. 2004	Yes	No	Yes	Yes	Yes	Yes	Yes	Yes	Yes	Include
Grygorowicz et al. 2017	Yes	Yes	Yes	Yes	Yes	Yes	Yes	Yes	Yes	Include
McCunn et al. 2017	Yes	Yes	Yes	Yes	Yes	Yes	Yes	Yes	Yes	Include
Armstrong & Greig 2018	No	No	No	No	No	No	No	No	No	Exclude
Chalmers et al. 2017	No	No	No	No	No	No	No	No	No	Exclude
Smith et al. 2017	No	No	No	No	No	No	No	No	No	Exclude
Lichtenstein et al. 2014	No	No	No	No	No	No	No	No	No	Exclude

### Meta-analysis of findings

For the sensitivity and specificity of screening tools and their accuracy, only three studies reported the estimates with 95% confidence intervals and were therefore suitable to be included in the meta-analysis. These studies are summarised in Table 5. There was considerably high intraclass correlation coefficient (ICC) for sensitivity and specificity (0.68, 95% confidence interval [CI] [0.52–0.84] and 0.64, 95% CI [0.61–0.66], respectively).

**TABLE 5a:** Sensitivity of included studies.

Study	Sensitivity (ICC)	Lower 95% CI limit	Upper 95% CI limit
Chorba et al. 2010	0.579	0.335	0.789
Padua et al. 2015	0.86	0.42	0.99
Grygorowicz et al. 2017	0.658	0.167	0.917

Note: The significance of the confidence interval is to generate a lower and upper limits for the mean. The large number (0.917) in Table 5a, as seen in Grygorowics et al. (2017) is combined with the small number (0.167) in Table 5a, as seen in Grygorowics et al. (2017) generates the interval estimate for mean. CI, confidence interval.

**TABLE 5b:** Specificity of included studies.

Study	Specificity (ICC)	Lower 95% CI limit	Upper 95% CI limit
Chorba et al. 2010	0.737	0.488	0.909
Padua et al. 2015	0.64	0.62	0.67
Grygorowicz et al. 2017	0.47	0.469	0.948

CI, confidence interval.

**FIGURE 2:**
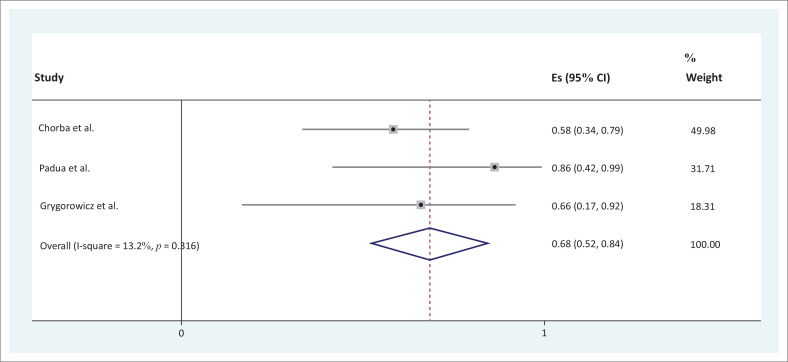
Forest plot graph for sensitivity.

**FIGURE 3:**
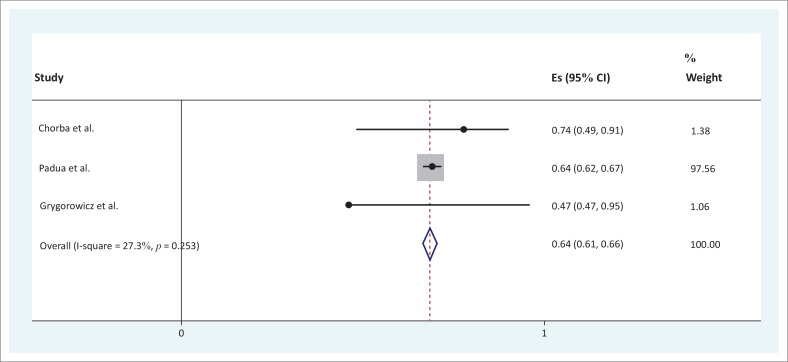
Forest plot graph for specificity.

In Figures 2 and 3, the horizontal axis on the forest plot represents the estimates with 95% confidence intervals of the included studies` sensitivity and specificity, respectively. Studies with wider confidence intervals were assigned a lower weighting. Grygorowicz et al. (2017) had a weighting of 18.31, Chorba et al. (2010) a weighting of 49.98 and Padua et al. (2015) a weighting of 31.71. The vertical line, namely the ‘line of null effect’, was used to interpret the significance of the statistic. The diamond represents overall estimate and confidence intervals when considering the combined results (Reid 2006). A significant overall estimate (sensitivity ICC 0.68, 95% CI [0.52–0.84] and specificity ICC 0.64 95% CI [0.61– 0.66]) of the combined studies was found as the diamond did not cross the line of null effect (Figures 2 and 3). To determine the proportion of variation across the studies, the *I*² statistic was used to quantify the heterogeneity from 1% to 100%. The heterogeneity was statistically insignificant (*p* = 0.316 and 0.253) as indicated by *I* ^2^ < 40% and the overlap of the 95% CIs on the forest plots (Figures 2 and 3).

### Narrative analysis

#### Screening tools

Four studies discussed FMS™ scores and tests as potential tools for predicting or preventing common soccer injuries. The FMS™ tools used by Silva et al. (2017) included test items such as the deep squat, hurdle step, in-line lunge, shoulder mobility, active straight-leg raise, trunk stability push-up, rotary stability, three clearing examinations, jump performance, instep kick speed (shot speed) and anaerobic performance to determine associations with FMS™ individual scores and overall FMS™ scores. The results suggest that FMS™ is suitable to determine the physical performance of soccer players and should not be used for downgrading their functional performance. This is because individual FMS™ scores may be a better determinant of performance than the FMS™ total score. In addition, the authors established minimal association between FMS™ scores and physical variables.

Lehance et al. (2009) compared pre-season muscular strength and power profiles in professional and junior elite soccer players, using functional performance, squat jump and 10-m sprints. The results showed that there was no significant difference in isokinetic muscle strength performance between the three groups in the study when considering normalised body mass parameters. Individual isokinetic profiles enabled the identification of 32 out of 57 (56%) participants presenting with lower limb muscular imbalance. Thirty-six out of 57 players were identified as having sustained a previous major lower limb injury. Of these 36 players, 23 still showed significant muscular imbalance (64%).

Hammes et al. (2016) showed that screening tools such as FMS™ are limited in predicting common soccer injuries in veteran football. The results showed that the potential risk factors for injuries in veteran football are age, lower body mass and a longer career.

To evaluate the inter- and intra-rater reliability of the test battery on a group of male elite soccer players, Frohm et al. (2012) used an FMS™ tool, which included the following items: one-legged squat, two-legged squat, straight-leg raise test, seated rotation test, in-line lunge test and active hip flexor test. The test battery was used to predict injuries caused by stability and mobility of the lower limb. The results of the study showed good inter-rater and intra-rater reliability and a strong need for future validation.

#### Accuracy of screening tools

Padua et al. (2015) showed that the LESS can be used to predict and prevent anterior cruciate ligament injuries, whilst Chorba et al. (2010) showed that anterior cruciate ligament rupture injuries can be predicted using the FMS™ tools. Grygorowicz et al. (2017) investigated conventional hamstring to quadriceps ratio to predict and prevent hamstring injuries. It was shown to be significant; however, because of the different threshold values, it was also shown to be highly biased. Gabbe et al. (2004) discussed musculoskeletal tests in his study, which demonstrated an excellent overall reliability (ICC 0.88–0.97) and an intra-rater reliability (ICC) of 0.63–0.99. The results showed that the sit and reach test, lumbar spine extension ROM, active hip internal ROM, active hip external ROM, passive straight-leg raise, active knee extension test and active slump test are reliable to use as pre-participation screening tools for sport participants.

To analyse the within-subject variation of the tuck jump screening assessment in elite male youth soccer players, Read et al. (2017) used the tuck jump assessment tool, which proved to be reliable in assessment. The assessments included elite male soccer players and showed that caution should be applied when solely interpreting the composite score because of the high within-subject variation in a number of individual criteria.

McCunn et al. (2017) assessed the intra- and inter-rater reliability of the SIMS using five sub-tests: the anterior reach (AR), single-leg deadlift (SLDL), in-line lunge (ILL), single-leg hop for distance (SLHD) and tuck jump (TJ). The action of the movement was filmed using an iPhone 4 device to obtain scores, which were compared individually as participants were blinded. The five sub-tests show to have acceptable landmarks with an odds ratio of 3.850 (CI 95% [0.980, 15.130]).

## Discussion

The pooled result from this review showed that there is a high sensitivity (ICC 0.68, 95% CI [0.52–0.84]) and high specificity (ICC 0.64, 95% CI [0.61–0.66]) amongst the included screening tools for preventing and predicting common injuries in soccer players. The screening tools included the FMS™, LESS and the conventional hamstring to quadriceps ratio. Chorba et al. (2010) showed that anterior cruciate ligament ruptures can be predicted using the FMS™ tool. The LESS, which is a variation of the drop jump test (DJT), used for evaluating landing patterns also emerged in this systematic review as a screening tool with high specificity and sensitivity. One of the included studies in the review (Padua et al. 2015) investigated the LESS as a screening tool for an anterior cruciate ligament injury-prevention programme in elite-youth soccer athletes. It shows that the LESS tool can be used to predict and prevent anterior cruciate ligament injuries. Studies on the accuracy of the LESS tool posit the tool as a valid and reliable screening tool for predicting and preventing soccer injuries irrespective of the skill sets of the rater. The construct validity of the LESS was established and the inter-rater reliability and intra-rater reliability of LESS were given as ICC = 0.84 and ICC = 0.91, respectively (Onate et al. 2010; Padua et al. 2009, 2011). Studies related to determining the accuracy of the LESS have largely been conducted by the same group of researchers; however, the emergent result from this systematic review is supportive of the conclusion that the LESS has high accuracy as a screening tool to predict and prevent common soccer injuries.

The findings of the study by Grygorowicz et al. (2017) on the use of conventional hamstrings to quadriceps ratio to predict and prevent hamstrings injuries amongst professional male soccer players were also significant. The findings were, however, biased because of the different threshold values. Therefore, caution must be exercised when utilising it for the purpose of predicting and preventing common soccer injuries and use in the clinical situation.

Similar to the meta-analysis for the accuracy of screening tools for common soccer injuries, the studies included in the qualitative discussion reported high accuracy of the included screening tools. The risk of bias shows 70% of studies with low risk and 30% with moderate risk.

Seven studies with a combined participant number of 335 were included in the objective to identify screening tools for soccer. These studies discussed functional screening scores and tests (FMS™) as potential tools for predicting and preventing common soccer injuries, but used different variations of the tests. The good reliability of the FMS™ could be the reason for its dominance amongst the included studies in this section of the systematic review. For example, Lehance et al. (2009) investigated the muscular strength, functional performances (squat jump and 10 metre sprint) and injury risk in professional and junior elite soccer players to compare pre-season muscular strength and power profiles in professional and junior elite soccer players. Studies show that the FMS™ has good reliability especially amongst American elite soccer players (Chimera & Warren 2016; Smith et al. 2013). An included study by Frohm et al. (2012) on reliability for a nine-test screening battery for male elite soccer players involved the use of FMS™ comprising one-legged squat, two-legged squat, straight-leg raise test, seated rotation test, in-line lunge test and active hip flexor test to predict injuries that are caused by stability and mobility of the lower limb. The study by Silva et al. (2017) included the deep squat, hurdle step, in-line lunge shoulder mobility, active straight-leg raise, trunk stability push-up and rotary stability, three clearing examinations, jump performance, in-step kick speed (shot speed) and anaerobic tests as part of functional assessment. The results from the study by Silva et al. (2017) suggest that FMS™ is suitable to determine the physical performance of soccer players for injury prediction and prevention. The study further suggested that FMS™, on the other hand, is not suitable for determining the weakness in the functional performance of soccer players as identified by individual FMS™ scores. Hammes et al. (2016), in determining the injury prediction in veteran football players using the FMS™, further strengthened the conclusion by Silva et al. (2017) that a screening tool like the FMS™ is limited to predict common soccer injuries especially amongst veteran soccer players.

## Grade of evidence

Ten key recommendations were observed for the class of evidence for our review according to Mueller et al. (2018): the protocol was developed at the initial stage of this systematic review; a research question was developed , followed by the study search strategy as defined in the protocol; the database to be searched and keywords were identified; the study eligibility had inclusion and exclusion criteria that were stated in the protocol; data extraction and the method of data extraction were performed by two blinded researchers; the study design was initially specified; the risk of bias was carried out; heterogeneity was observed; statistics were undertaken. Publication bias, however, needs to be considered as studies were only included from the year 2000–2018.

Considering the five domains of the GRADE system (Guyatt et al. 2008) and the types of studies included in the meta-analysis, there is only level 2 evidence available on the accuracy of screening tools to predict common soccer injuries.

## Limitations and recommendations

The limitation of our study is based on the extraction of data from included studies. Most studies did not give the specificity and sensitivity of screening tools, and therefore only a limited number of three studies were included in the meta-analysis. It does however indicate the need for studies to determine the accuracy of screening tools.

It is also important to note that although evidence exists on screening tools for injuries, many of the studies were generic and not focused on soccer injuries. Many of the available studies also have low methodological quality, which prevented many of them from meeting the inclusion criteria of our study. Hence, there is a need for sport professionals and scholars to focus more on conducting well-designed studies to determine the accuracy of screening tools for common soccer injuries.

## Conclusion

The screening tools assessed for the prediction of common soccer injuries that emerged from this systematic review include the FMS™, the LESS, the Tuck Jump Assessment, the Soccer Injury Movement Screening (SIMS) and the conventional hamstrings to quadriceps ratio with high evidence of predicting common soccer injuries. These tools were of high sensitivity and specificity and can thus be recommended for use in clinical practices.

## Figures and Tables

**FIGURE 1-A1:**
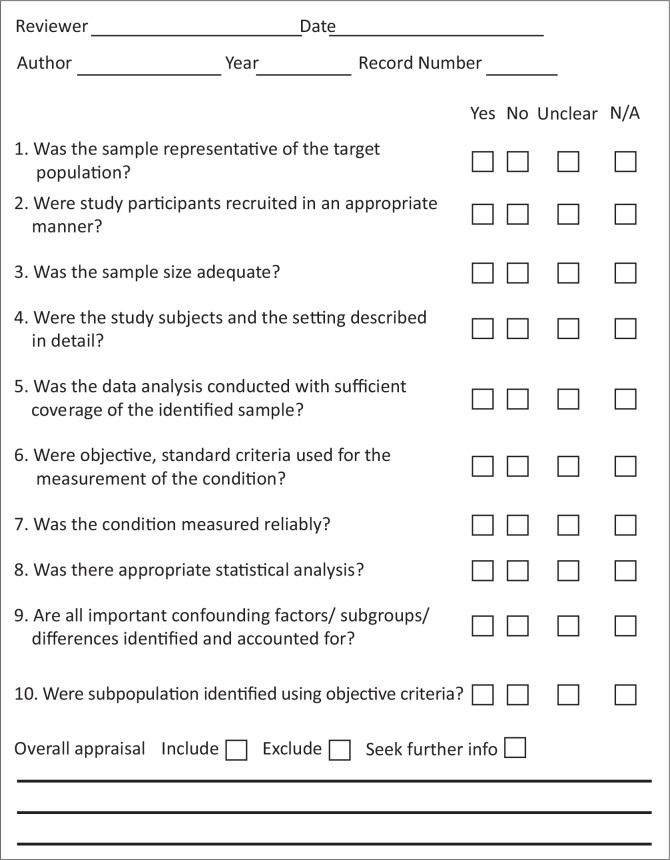
Joanna Briggs Institute (JBI) critical appraisal checklist for studies reporting prevalence data.
